# Serum biomarkers for neurofibromatosis type 1 and early detection of malignant peripheral nerve-sheath tumors

**DOI:** 10.1186/1741-7015-11-109

**Published:** 2013-04-23

**Authors:** Su-Jin Park, Birgit Sawitzki, Lan Kluwe, Victor F Mautner, Nikola Holtkamp, Andreas Kurtz

**Affiliations:** 1 Berlin-Brandenburg Center for Regenerative Therapies, Charité - Universitätsmedizin Berlin, Augustenburger Platz 1, Berlin, 13353, Germany; 2 Institute for Clinical Immunology, Charité - Universitätsmedizin Berlin, Augustenburger Platz 1, Berlin, 13353, Germany; 3 Experimental Tumor Research, Phakomatoses, Department of Neurology, Universitätsklinikum Hamburg-Eppendorf, Martinistrasse 52, Hamburg, 20246, Germany; 4 College of Veterinary Medicine, Seoul National University, 599 Gwanangno, Gwanak-gu, Seoul, 151-742, Republic Korea

**Keywords:** Neurofibromatosis type 1, Serum biomarker, Antibody array, Cytokines, Malignant peripheral nerve-sheath tumor

## Abstract

**Background:**

Neurofibromatosis type 1 (NF1) is a hereditary tumor syndrome characterized by the development of benign nerve-sheath tumors, which transform to malignant peripheral nerve-sheath tumors (MPNST) in about 8 to 13% of patients with NF1. MPNST are invasive sarcomas with extremely poor prognosis, and their development may correlate with internal tumor load of patients with NF1. Because early identification of patients with NF1 at risk for developing MPNST should improve their clinical outcome, the aim of this study was to identify serum biomarkers for tumor progression in NF1, and to analyze their correlation with tumor type and internal tumor load.

**Methods:**

We selected candidate biomarkers for NF1 by manually mining published data sources, and conducted a systematic screen of 56 candidate serum biomarkers using customized antibody arrays. Serum from 104 patients with NF1 with and without MPNST, and from 41 healthy control subjects, was analyzed. Statistical analysis was performed using the non-parametric Mann–Whitney *U*-test, followed by Bonferroni correction.

**Results:**

Our analysis identified four markers (epidermal growth factor receptor, interferon-γ, interleukin-6, and tumor necrosis factor-α) for which significantly different serum concentrations were seen in patients with NF1 compared with healthy controls. Two markers (insulin-like growth factor binding protein 1 (IGFBP1) and regulated upon activation, normal T-cell expressed and secreted (RANTES)) showed significantly higher concentrations in patients with NF1 and MPNST compared with patients with NF1 without MPNST. A correlation with internal tumor load was found for IGFBP1.

**Conclusion:**

Our study identified two serum markers with potential for early detection of patients with NF1 at risk for developing MPNST, and four markers that could distinguish between patients with NF1 and healthy subjects. Such markers may be useful as diagnostic tools to support the diagnosis of NF1 and for timely identification of MPNST. Moreover, the data suggest that there is a systemic increase in inflammatory cytokines independently of tumor load in patients with NF1.

## Background

Neurofibromatosis type 1 (NF1) is an autosomal dominant tumor syndrome, with an estimated incidence at birth of 1 in 2500 [1] and complete penetrance. NF1 is caused by mutations in the *NF1* gene [2,3], coding for the tumor-suppressor protein neurofibromin, which acts as a Ras-negative regulator via its Ras-GTPase activating protein (GAP) domain. Monoallelic and biallelic loss of *NF1* leads to increased Ras activity in affected cells.

Among the defining features of NF1 is the development of benign peripheral nerve-sheath tumors, which can arise at virtually any site in the body. Whereas cutaneous neurofibromas (CNF) are mostly visible and palpable, subcutaneous neurofibromas, internal plexiform neurofibromas (PNF) and malignant peripheral nerve-sheath tumors (MPNST) are difficult to detect, quantify, or monitor [4].

MPNST are the major cause for the reduced life span of patients with NF1, and they will lead to death if not detected early and treated in time. The primary forms of treatment are selective resection of benign PNF, and radical surgical resection of MPNST [5-8]. However, the invasive growth pattern of MPNST frequently prohibits complete tumor removal, especially when diagnosed late in their development. Moreover, although chemotherapy and radiotherapy may delay recurrence, they have little effect on long-term survival [7,9].

The lifetime risk of MPNST for patients with NF1 patients has been estimated to be about 8 to 13% and thus is more than 1000 times higher for these patients than for the general population. Moreover, many patients with NF1 develop MPNST at the unusually young age of around 30 years [10,11], compared with the median age of diagnosis of 62 years in the general population [12]. Because MPNST develop by malignant progression of pre-existing PNF, the risk to develop an MPNST increases to almost 50% in patients with NF1 and PNF [12,13].

It is possible to detect dermal and superficial neurofibromas directly by optical or ultrasonography methods [14], whereas PNF and MPNST are often diagnosed only after clinical symptoms occur. Systematic analysis of the internal tumor load of patients with NF1 by whole-body magnetic resonance imaging (MRI) suggests an association between the risk for MPNST development and internal PNF tumor load [15]. However, these imaging techniques are not applicable as a routine screening tool. The search for surrogate biomarkers for timely identification of patients at risk for malignant transformation has mostly been based on the assumption that overexpression of proteins in PNF and MPNST subsequently leads to increased systemic concentrations [16-19]. Among such factors, serum levels for midkine and for stem cell factor were found to be significantly increased in a cohort of 39 patients with NF1, although no correlation with tumor load or MPNST was found [20]. Recently, we identified melanoma-inhibitory activity (MIA; also known as cartilage-derived retinoic acid-sensitive protein (CD-RAP)) as a marker for the internal tumor load in a cohort of 42 patients with NF1 [21]. MIA was shown previously to be a biomarker for malignant neuroectotermal tumors [22]. In another study, 92 genes encoding putative secreted proteins in neurofibromas and MPNST were analyzed for their potential as serum markers [23]. Of these, only adrenomedullin (ADM) was confirmed as differentially expressed and increased in the serum of patients with NF1, and serum concentrations were found to be even higher in a small sample of patients with MPNST (n = 5).

Tumorigenesis in NF1 is strongly influenced by the haploinsufficient *NF1+/−* systemic environment, which may also promote invasion of PNF and MPNST by *NF1+/−* monocytes and mast cells [24-30]. Therefore, we included immunomodulating cytokines in the present screen for serum biomarkers, in addition to factors secreted by tumor cells in PNF and MPNST. Of the 56 candidate proteins analyzed, we identified four proteins with significantly altered serum concentrations in patients with NF1 compared with non-NF1 control subjects, but independently of tumor load. Two proteins were significantly increased in patients with MPNST, and one of these also correlated with internal tumor load.

## Methods

### Ethics approval

The study was approved by the internal review board (Ethics Committee of the Ärztekammer Hamburg number OB-089/04) in compliance with the Declaration of Helsinki, and informed consent was obtained before sample collection.

### Patients and serum collection

Serum samples from patients with NF1 were obtained from the Department of Maxillofacial Surgery (University Hospital Eppendorf, Hamburg, Germany). All patients with NF1 were clinically diagnosed according to published guidelines and criteria [31]. Serum samples from healthy control subjects were obtained from the Institute of Medical Immunology (Charité - Universitätsmedizin Berlin) from anonymized leftover diagnostic samples. For detailed information on the patient cohorts, see Additional file 1. Venous blood (1 to 10 ml) was collected, then separated by centrifugation within 2 hours of collection, and serum samples were immediately frozen in aliquots and stored at −80°C until use. Fresh aliquots were used for each analysis.

### Candidate marker selection

**Figure 1:**
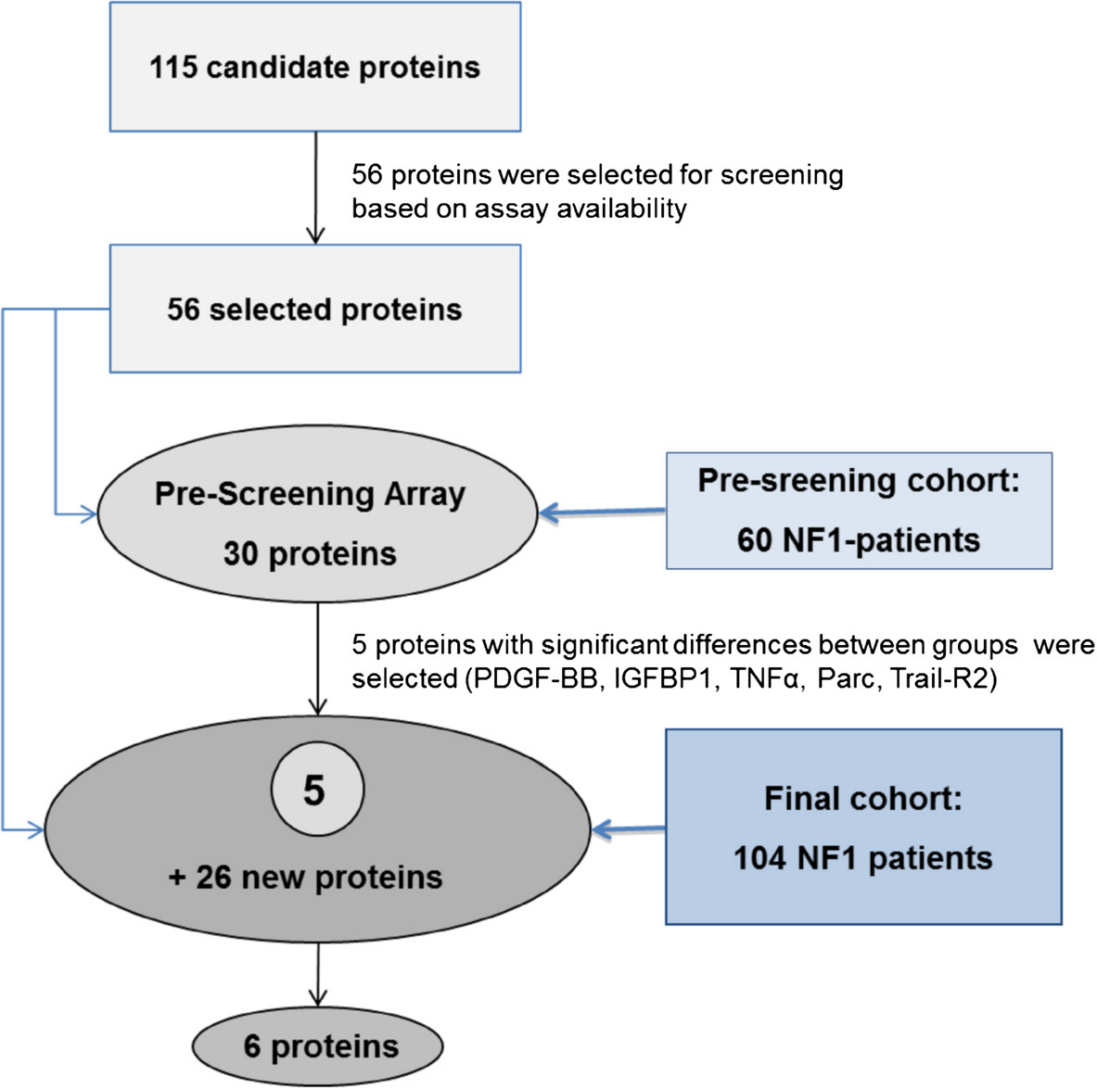
**Schematic outline of the candidate marker selection and screening procedures.** The initial 115 possible serum markers for neurofibromatosis type 1 (NF1) tumors consisted of proteins that are overexpressed in plexiform neurofibromas (PNF) or malignant peripheral nerve-sheath tumors (MPNST), have been found to promote tumor growth (n = 79), or are immunmodulatory cytokines (n = 36). These 115 proteins were selected by manual searches of published data. Of the 115 initially selected proteins, 56 were screened in two steps, with sera from 60 patients with NF1 in the first pre-screening step, and from 104 patients with NF1 in the second. Five proteins (platelet-derived growth factor (PDGF)-BB, insulin-like growth factor binding protein (IGFBP)1, tumor necrosis factor (TNF)-α, macrophage inflammatory protein (MIP)-4 and TNF-related apoptosis inducing ligand (TRAIL)-R2) were selected from the pre-screening step and included in the main screening of 104 NF1 sera, which confirmed IGFBP1 and TNF-α, and identified four new proteins (interleukin (IL)-6, interferon (IFN)-γ, epidermal growth factor receptor (EGFR), and Regulated upon activation, normal T-cell expressed and secreted (RANTES)) as potential biomarkers.

Selection of candidate markers was based on a manual literature search of publications and publicly available databases describing 1) protein levels in serum, plasma, or cell supernatants from patients with nervous system or epithelial tumors or from cell lines, or 2) differential gene expression between the normal peripheral nervous system, neurofibroma, and MPNST. and 3) immunomodulatory cytokines (see Additional file 2). The list of identified candidate factors was further reduced by selecting factors with known functional roles in tumorigenesis such as growth promotion, migration and metastasis, angiogenesis, and immune modulation, based on information from the Gene Ontology and GeneCards databases [32,33]. The final selection of candidate factors was based on the availability of suitable screening platforms. Of the 115 initially identified potential serum proteins, a list of 56 candidate factors was compiled for screening of serum samples based on the availability of antibodies for customized array analysis (Figure 1, see Additional file 2).

### Serum screening

Customized human cytokine arrays (Quantibody; RayBiotech Inc., GA, USA) were used to determine serum protein concentrations. Analyses were performed in accordance with the manufacturer’s instructions. Imaging was performed using the accompanying software (Quantibody Array Testing Software; RayBiotech Inc.). Potential marker proteins were initially identified by screening of 30 candidate proteins using 60 NF1 sera (n = 27, n = 13, and n = 20, respectively, for patients with NF1 with PNF, with MPNST, and without tumors) and 20 control sera. Secondary screening was performed on the five proteins that showed significant differences in the pre-screening round (platelet-derived growth factor (PDGF)-BB, insulin-like growth factor binding protein (IGFBP)1, tumor necrosis factor (TNF)-α, macrophage inflammatory protein (MIP)-4, TNF-related apoptotic ligand (TRAIL)-R2), together with another set of 26 candidate proteins (see Additional file 2). In the second round, 104 NF1 sera and 41 control sera were screened. Altogether, 56 candidate proteins were screened, and 104 NF1 and 41 control sera were used. The candidate proteins were simultaneously scanned by multiplex detection in quadruplicate spots per array. Hence, all sera were analyzed in at least quadruplicates. A flowchart of the screening procedure is provided (Figure 1). Serum factors with significantly different levels between groups (with the exception of epidermal growth factor receptor (EGFR)) were verified in a limited subset of NF1 (n ≥ 11) and control (n ≥ 5) serum samples using ELISA for IGFBP1 (Abcam, Cambridge, UK), and cytometric bead array (CBA) (BD Bioscience, Heidelberg, Germany) for RANTES (regulated upon activation, normal T-cell expressed and secreted), interferon (IFN)-γ, interleukin (IL)-6 and TNF-α. The analyses were performed in accordance with the manufacturers’ instructions. Capture beads were analyzed on a flow cytometer (FACSCalibur, BD Biosciences, Heidelberg, Germany), and flow-cytometry data were evaluated with FCAP Array analysis software (Soft Flow Inc., MN, USA) (see Additional file 3).

### Statistical analysis

Serum levels of the candidate markers in the NF1 patient group and control group were analyzed with respect to median levels and interquartile ranges. To verify all data for normal distribution, the Kolmogorov-Smirnov test was used. Stratified patient groups were compared using the Mann–Whitney *U*-test for continuous non-parametric variables. For assessing the discriminatory power of individual markers, the receiver operating characteristic (ROC) curve and area under the curve (AUC) were calculated. For significance testing, the non-parametric Mann–Whitney *U*-test followed by Bonferroni correction was used. Two-tailed tests were used for all analyses. *P*<0.05 was considered significant. Statistical analysis was performed using SPSS version 18 software (SPSS, Inc., IL, USA) and GraphPad Prism software (version 5.0 GraphPad Software Inc., CA, USA).

## Results and discussion

In the present study, we used antibody arrays to identify serum biomarkers for NF1 in general and for NF1-associated nerve-sheath tumors in particular. Manual data mining identified 115 proteins as potential serum markers for NF1. Of these 115 proteins, 79 are expressed in PNF or MPNST, or have been described as tumorigenic serum factors. The other 36 proteins are immunomodulatory cytokines. These proteins were selected because of evidence that systemic *NF1* haploinsufficiency in patients with NF1 may result in overexpression of cytokines [34,35] (see Additional file 2). We reasoned that the degree of immunological deregulation may indirectly signal increased risk for tumor growth and malignant transformation. The sera of 104 patients with NF1 with different tumor types, and 41 matched control subjects (Table 1; see Additional file 1) were analyzed, and 56 of the 115 initially identified candidate proteins were screened (see Additional file 2). Pre-screening was carried out with 60 sera (comparing controls, NF1 without PNF or MPNST, NF1 with PNF, and NF1 with MPNST), using an array of 30 proteins (see Additional file 1), and this identified 5 proteins with significantly increased levels in serum of patients with NF1. When testing for these 5 proteins was performed for in the complete cohort of 104 patients, only 2 proteins (IGFBP1 and TNF-α) were confirmed to be significantly different in NF1 sera. We also screened for another 26 proteins in the complete cohort and found significant differences for 6 proteins (Figure 1).

**Table 1:** Characteristics of patient cohorts recruited for the study

	**Controls**	**NF1 patients**	**NF1 patients**	**NF1 patients**
		**w/o PNF w/o MPNST**	**with PNF w/o MPNST**	**with MPNST**
**n**	41	35	39	30
**mean age in years**	47 (range 24–66)	32 (range 14–48)	34 (range 15–63)	34 (range 16–62)
♀**/**♂	22/19	22/13	17/22	17/13
**whole body MRI**		25/35	33/39	30/30

Serum concentrations of all six candidate markers were independent of age and sex in the tested population (mean age was 46 and 32 years for the healthy controls and the NF1 group, respectively). This is important, as circulating levels of the inflammatory cytokines TNF-α and IL-6 may increase with age [36,37].

**Figure 2:**
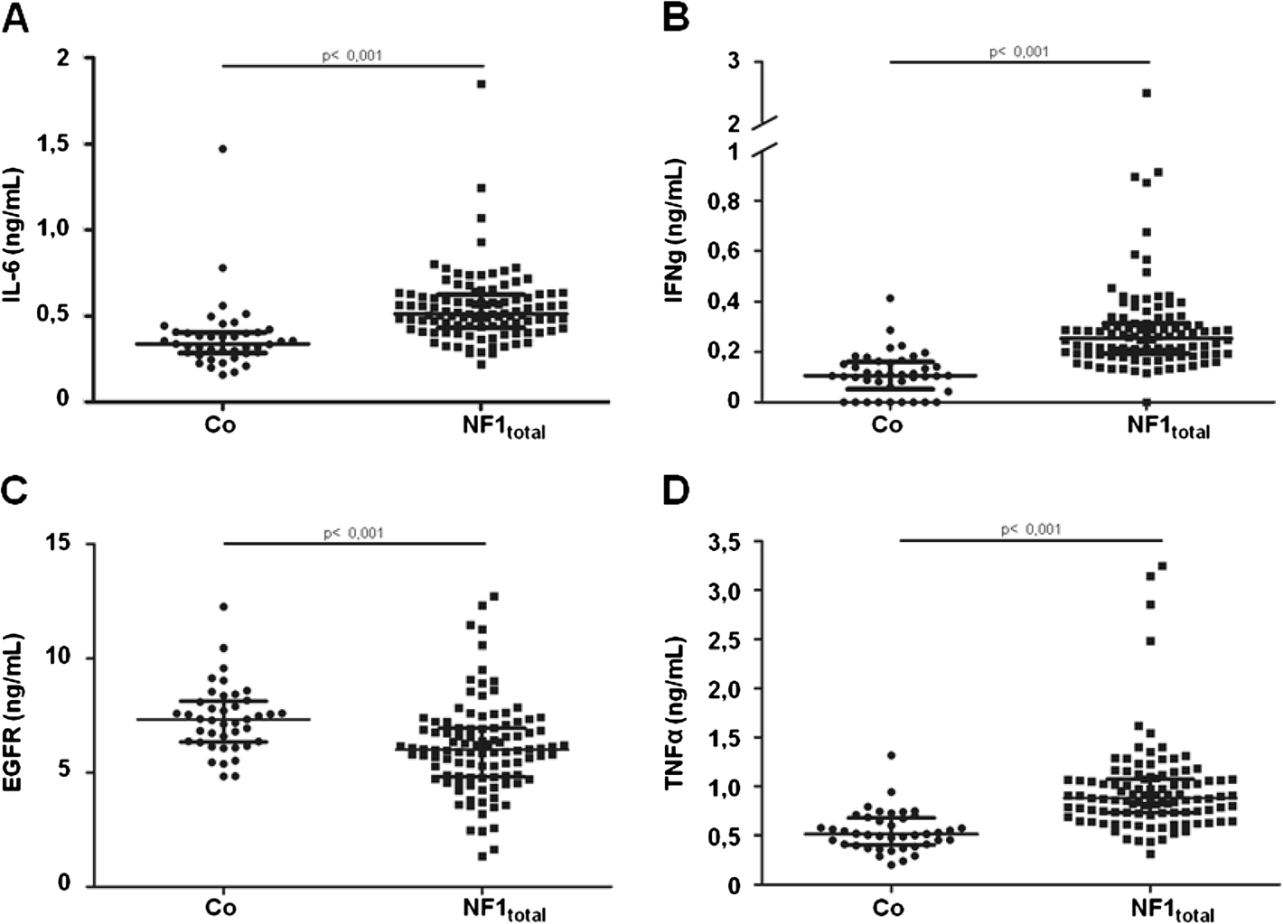
**Quantitative protein array results of sera from 104 patients with neurofibromatosis type 1 (NF1) (the total group; NF1**_**total**_**) compared with 41 healthy controls (Co).** Differences were highly significant for: **(A)** interleukin (IL)-6, **(B)** interferon (IFN)-γ, **(C)** epidermal growth factor receptor (EGFR), **(D)** and tumor necrosis factor (TNF)-α. None of the other proteins tested showed significant differences between the two groups. Statistical analysis was performed using the non-parametric Mann–Whitney *U*-test, including Bonferroni correction.

Significant differences in serum concentration were found between patients with NF1 and healthy subjects for four proteins (Figure 2). The serum concentration of EGFR was significantly lower and the serum concentrations of the inflammatory cytokines IFN-γ, TNF-α and IL-6 were significantly higher in patients with NF1 compared with healthy subjects.

**Figure 3:**
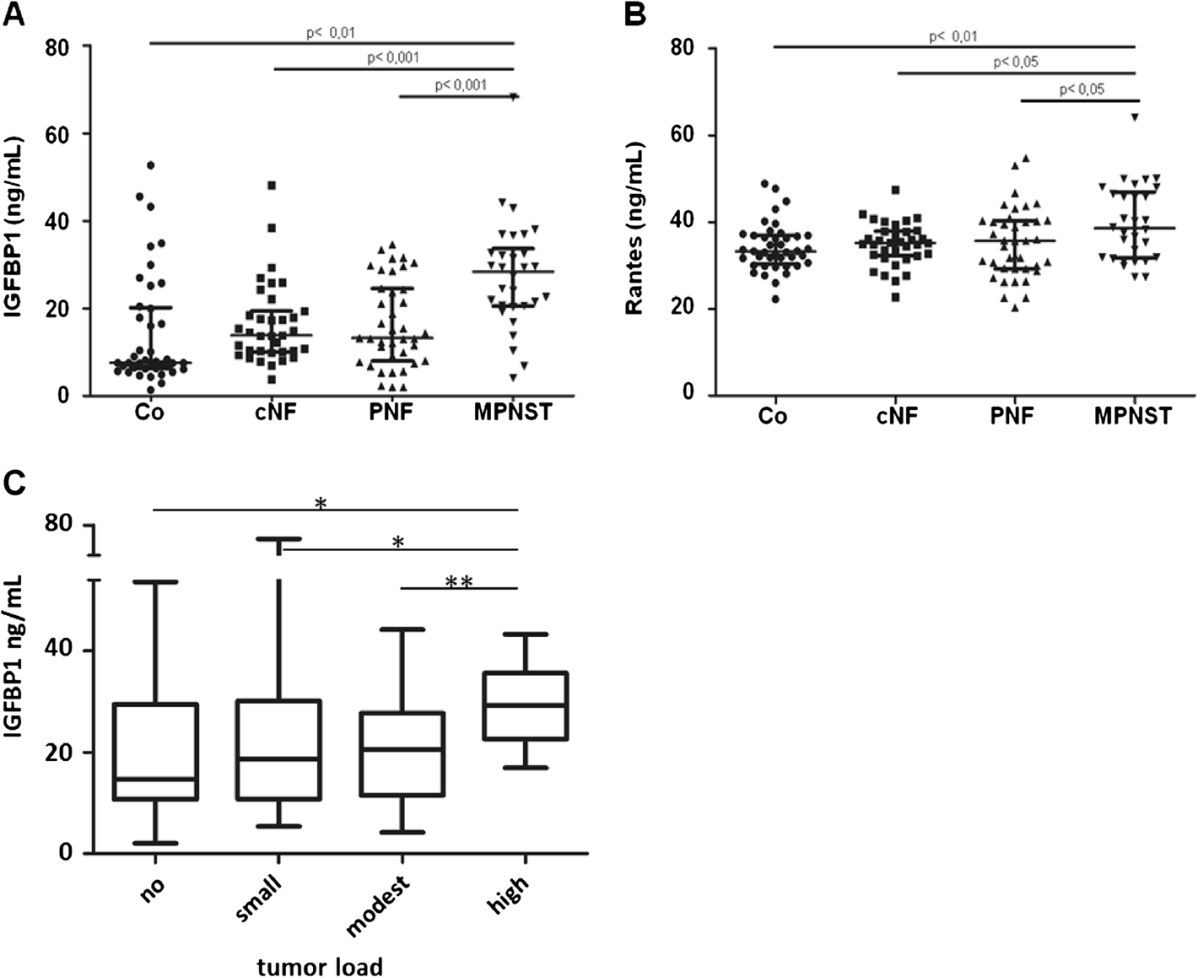
**Quantitative protein array results of sera from 41 healthy controls and 104 patients with neurofibromatosis type 1 (NF1) subdivided into three groups.** These comprised 35 patients with NF1 with no plexiform neurofibromas (PNF) and no malignant peripheral nerve-sheath tumors (MPNST) (cutaneous neurofibromas; cNF), 39 patients with NF1 with PNF and no MPNST, and 30 patients with NF1 with MPNST. **(A)** Insulin-like growth factor binding protein (IGFBP)1 and **(B)** Regulated upon activation, normal T-cell expressed and secreted (RANTES). **(C)** IGFBP1 serum concentrations in patients with NF1 with different internal tumor loads as measured by MRI-based volumetry (0 cm^3^ = no load, 1 to 99 cm^3^ = small load; 100 to 500 cm^3^ = modest load; >500 cm^3^ = high load). Statistical analysis was performed using the non-parametric Mann–Whitney *U*-test, including Bonferroni correction (*P≤0.05; **P≤0.01; ***P≤0.001).

Further stratification of the NF1 cohort into three clinical groups (patients with NF1 with 1) CNF only, 2) with PNF and 3) with MPNST) (Table 1) identified two more proteins, IGFBP1 and RANTES, for which there were significant differences between patients with NF1 with MPNST and those without MPNST. Of note, no difference was detected between the control group and patients with NF1 without MPNST (n = 74) for these two proteins (Figure 3A,B).

A previous study using volumetric analysis of whole-body MRI data for patients with NF1 indicated a correlation between internal tumor load and risk for malignant transformation of PNF into MPNST [4]. Therefore, we attempted to correlate the serum concentration of the six identified serum biomarkers with internal tumor load for the 87 patients with NF1 for which these data were available (see Additional file 1). Importantly, the serum concentrations of IGFBP1, but not of any of the other five markers, correlated with internal tumor load (Figure 3C).

This finding is in line with the correlation between IGFBP1 serum levels and presence of MPNST (Figure 3A), and further identifies IGFBP1 as a potential risk marker for malignant transformation. The data also suggest that increased cytokine levels in patients with NF1 are independent of tumor load. Rather, these results imply that systemic NF1 haploinsufficiency triggers a permanent and systemic inflammatory status in patients with NF1, which is reflected by a significant increase in IFN-γ, TNF-α and IL-6 [34].

Protein array data were confirmed in a small subgroup by CBA and ELISA (see Additional file 3) for IFN-γ, TNF-α, IL-6, IGFBP1 and RANTES. We did not reassess the level of EGFR because of its comparably lower AUC.

**Figure 4:**
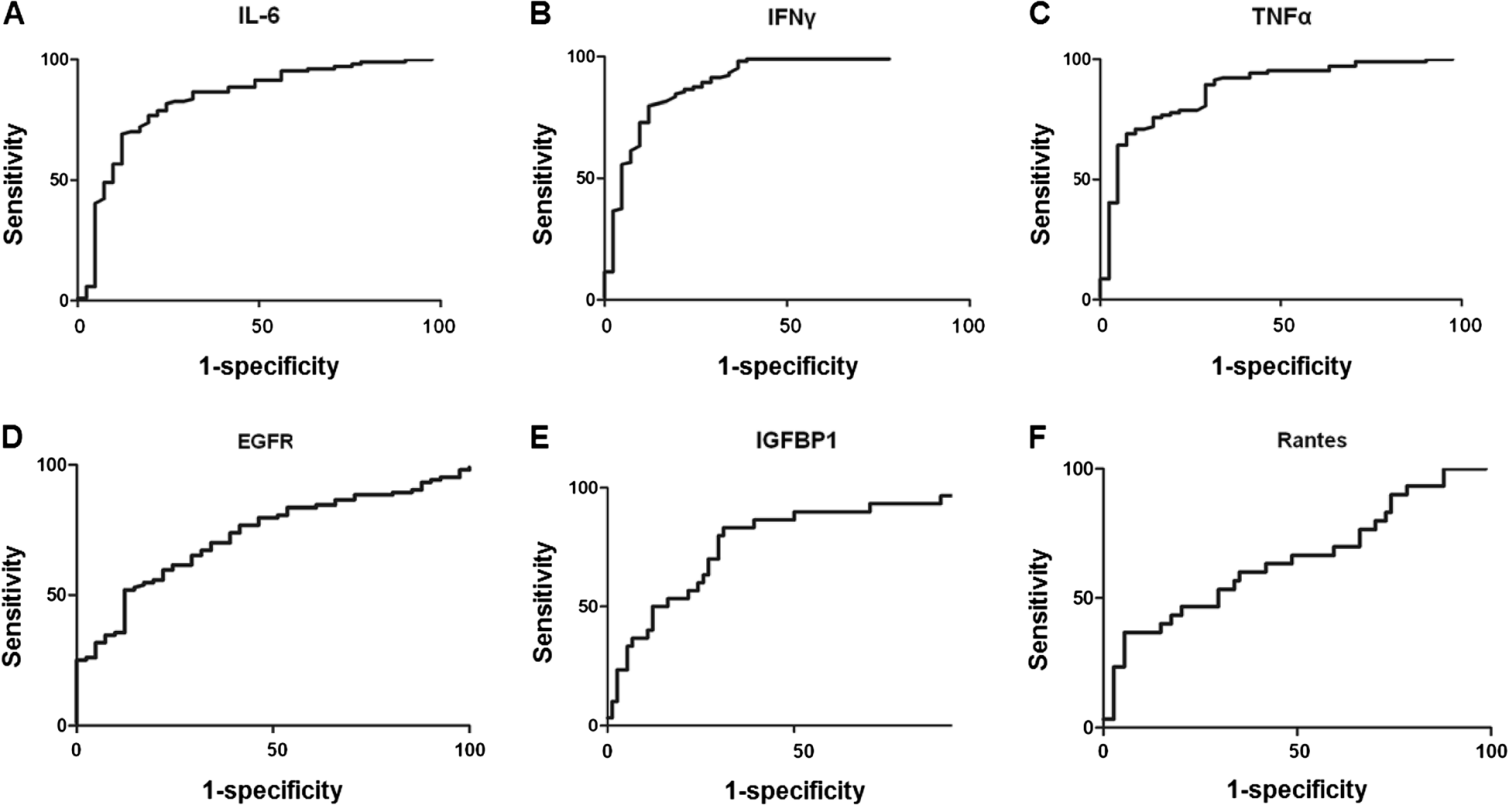
**Discrimination between the neurofibromatosis type 1 (NF1) group and control group receiver operating characteristic (ROC) curves. (A)** interleukin (IL)-6, **(B)** interferon (IFN)-γ, **(C)** tumor necrosis factor (TNF)-α and **(D)** epidermal growth factor receptor (EGFR). The ROC curves for discrimination between patients with NF1 with and without malignant peripheral nerve-sheath tumors (MPNST) are shown in **(E)** for insulin-like growth factor binding protein (IGFBP)1 and **(F)** for Regulated upon activation, normal T-cell expressed and secreted (RANTES).

The diagnostic potential of the factors we identified was determined by computing the AUC of the individual ROC curves. Specificity was determined at a sensitivity of 90% (Table 2; Figure 4). For all six candidates the AUC was significant (*P*<0.05). The largest AUC for the NF1 markers was found for IFN-γ (0.90), followed by TNF-α (0.88), IL-6 (0.83) and EGFR (0.73). The increased levels of pro-inflammatory cytokines did not depend on tumor load, as often found for patients with other tumors [38]. Rather, our data showed an increased systemic pro-inflammatory state in patients with NF1 compared with non-NF1 controls, supporting our assumption that increased cytokine levels in NF1 are caused by the NF1+/− environment. Whether this is due to an increase in mast cells and monocyte activity, or to other generalized changes in the immune status of these patients, remains unclear [35,39].

**Table 2:** Overview on serum marker features at 90% sensitivity

	**NF1 marker**				**MPNST marker**	
	**IFN-γ**	**EGFR**	**IL-6**	**TNF-α**	**IGFBP1**	**RANTES**
**Sensitivity:**	90,4	90,4	90,4	90,4	90,0	90,0
**Specificity:**	70,7	14,6	51,2	68,3	50,0	25,7
**NPV**	88,0	60,3	84,2	87,7	79,3	72,0
**PPV**	75,5	51,4	64,9	74,0	65,9	58,1
**cut off (ng/ml):**	0,15	8,57	0,34	0,59	13,77	30,72

The prevalence for NF1 markers was set at 0.5, while the prevalence for MPNST markers was set at 10%. The risk of NF1 patients to develop an MPNST is 8-13%.

In patients with MPNST, the AUC of IGFBP1 (0.77) was larger than that of RANTES (0.65) (Figure 4). RANTES is an inflammatory chemokine known to mediate chemotactic activity in immune cells such as T cells and monocytes [40]. RANTES was also shown to be expressed by breast carcinomas [41], and correlated with a more advanced stage of disease, suggesting a role for cancer progression. Increased serum levels of RANTES and IGFBP1 may be the result of increased secretion by the tumor cells themselves, or by immune cells in response to the neoplastic process, or by both mechanisms.

IGFBP1 binds IGF-I and IGF-II, and prolongs their half-life. Plasma levels of IGFBP1 are regulated by hormones outside of the growth-hormone axis, including insulin, glucagon, and cortisol [42,43]. An inverse correlation has previously been indicated between IGFBP1 levels and carcinogenesis [44,45]. The expression of IGF-I and growth-hormone receptors in PNF and MPNST in patients with NF1, and the correlation between IGF-I receptor levels and the increased mitosis index of PNFs, suggest sensitivity of these tumors to IGFBP1-regulated factors [10,46]. Taken together, IGFBP1 may modulate IGF access to PNF and MPNST, although this mechanism still needs to be elucidated.

The reasons for the reduced circulating EGFR levels that we detected in patients with NF1 are unclear. A possible functional explanation may be enhanced survival of cells that retain their EGFR on the cell surface, providing readiness for EGF signaling [47]. Similarly, EGF signaling has been shown to enhance tumorigenesis in NF1 animal models, and NF1-derived Schwann cells and fibroblasts are highly sensitive to EGF [48]. Hence, it seems that retaining the EGFR on the cell surface leads to reduced circulation of soluble EGFR, and provides an environment that promotes tumorigenesis, as seen in patients with NF1.

Recently, two studies identified MIA and ADM as potential NF1 tumor markers in cohorts of 42 and 32 patients, respectively [21,23]. There was also a trend towards correlation between ADM and MPNST, although the MPNST group was too small to show significance (n = 5). MIA concentration was particularly high in patients with NF1 with either PNF or large numbers of neurofibromas, and correlated with internal tumor burden. Both of these factors seem to be related to tumor burden in NF1, although induction as a result of changed systemic environment due to haploinsufficiency cannot be excluded. It would be intriguing to investigate further what role, if any, a systemic inflammatory environment may play in the early stages of tumorigenesis in patients with NF1.

## Conclusions

Our study encompasses the largest cohort of patients with NF1 (n = 104) screened to date for potential serum markers in this rare genetic cancer syndrome. We identified four potential biomarkers, which may assist in the diagnosis of NF1, and two further markers (IGFBP1 and RANTES) that correlate with the presence of MPNST. Intriguingly, IGFBP1 also seems to correlate with internal tumor burden, and thus may indicate increased risk for malignant transformation in patients with NF1. Furthermore, our data reveal a systemic pro-inflammatory profile in patients with NF1, which is probably caused by *NF1* haploinsufficiency. Serum biomarkers that could aid in the early detection of malignant progression would be extremely helpful because therapeutic interventions could be initiated before further spread of the tumor or development of metastasis takes place. Both the current and previous data are very promising for further validation of the data in even larger cohorts. It would be intriguing to further investigate what, if any, role a systemic inflammatory environment may play in the early stages of tumorigenesis in patients with NF1. Multicenter studies in larger cohorts will be necessary to validate the identified markers, and to elucidate a possible role of inflammatory cytokines in tumorigenesis.

## Abbreviations

ADM: Adrenomedullin; AUC: Area under the curve; CBA: Cytometric bead array; CNF: Cutaneous neurofibromas; EGFR: Epidermal growth factor receptor; ELISA: enzyme-linked immunosorbent assay (ELISA); IFN-γ: Interferon-γ; IGF-1: Insulin-like growth factors; IGFBP1: Insulin-like growth factor binding protein 1; IL-6: Interleukin 6; MIA: Melanoma-inhibitory activity/cd-rap; MIP-4: Macrophage inflammatory protein-4; MK: Midkine; MPNST: Malignant peripheral nerve-sheath tumors; MRI: Magnetic resonance imaging; MRT: Magnetic resonance tomography; NF1: Neurofibromatosis type 1; PDGF-BB: Platelet-derived growth factor-BB; PNF: Plexiform neurofibromas; RANTES: Regulated upon activation, normal T-cell expressed and secreted; ROC: Receiver operating characteristic; TNF-α: Tumor necrosis factor-α; TRAIL-R2: TNF-related apoptosis inducing ligand-R2.

## Competing interests

The authors have no competing interests to declare.

## Authors’ contributions

SP performed array and immunoassay experiments, and analyzed the data. BS advised on the choice of candidate markers, and supervised analytical experiments and analysis. VM collected and provided clinical data and specimens, and advised on experimental design. LK collected and selected serum samples for analysis, and contributed to data acquisition of data; NH coordinated the study, evaluated and interpreted the data, and drafted the manuscript. AK conceived the hypothesis, evaluated data, and critically revised the manuscript. All authors approved the final version of the manuscript.

## Pre-publication history

The pre-publication history for this paper can be accessed here:

http://www.biomedcentral.com/1741-7015/11/109/prepub

## Supplementary Material

Additional file 1 **List and detailed information of patient and control cohorts used in the study.** Abbreviations: nd, not done. Click here for file

Additional file 2 **List of candidate markers selected by manual curation of published data and text.** The proteins used in the screenings are shown in bold and italic [49-63]. Click here for file

Additional file 3 **Reassessment of protein serum markers interferon (IFN)-γ, interleukin (IL)-6, tumor necrosis factor (TNF)-α, insulin-like growth factor binding protein (IGFBP) and Regulated upon activation, normal T-cell expressed and secreted (RANTES) by cytometric bead array (CBA) and ELISA (arbitrary serum concentration units).** Between 11 and 15 randomly selected sera from the different NF1 groups (for IFN-γ, IL-6, TNF-α: all NF1 vs. control; for IGFBP and RANTES: NF1 with no PNF or MPNST, NF1 with only PNF- and NF1 with MPNST) and 5 control sera were tested as indicated (ND, not determined). Where available, associated protein array data are shown. Statistical analysis is shown for CBA/ELISA data (*t*-test). Click here for file
