# Supplementary material for: Serum biomarkers for neurofibromatosis type 1 and early detection of malignant peripheral nerve-sheath tumors
Source: BMC Med. 2013 Apr 23;11:109. doi: 10.1186/1741-7015-11-109 (PMC3648455; doi:10.1186/1741-7015-11-109)
Supplement: Additional file 1 — List and detailed information of patient and control cohorts used in the study. Abbreviations: nd, not done. [file 1741-7015-11-109-S1.pdf]

**Additional File 1:** List and detailed information of patient- and control cohorts used in the study.

| No. | Age | Gender | Subcutaneous NF | Cutaneous NF | PNF | MPNST | Tumorvolume ($cm^3$) |
|---|---|---|---|---|---|---|---|
| 1 | 20 | f | none | none | 1 | | 0 |
| 2 | 30 | f | none | 100 | none | | 0 |
| 3 | 40 | f | 3-9 | 3-9 | none | | 0 |
| 4 | 41 | f | 3 to 9 | none | none | | 0 |
| 5 | 32 | f | none | 10-50 | none | | 0 |
| 6 | 29 | m | 600 | none | none | | 0 |
| 7 | 39 | f | 100 | 1000 | >2 | | 0 |
| 8 | 15 | m | 60 | 10-50 | 2 | | 0 |
| 9 | 26 | m | 10-50 | 150 | 1 | | 0 |
| 10 | 22 | m | 2 | none | none | y | 0 |
| 11 | 26 | f | 2 | 10-50 | none | | 0 |
| 12 | 36 | m | 10-50 | 10-50 | none | | 0 |
| 13 | 9 | m | none | none | none | | 0 |
| 14 | 21 | f | 10 to 50 | 2 | none | | 0 |
| 15 | 39 | f | none | 10 to 50 | 2 | | 0 |
| 16 | 34 | m | 10-50 | 10-50 | none | | 0 |
| 17 | 25 | m | 3-9 | 3-9 | 1 | | 0 |
| 18 | 15 | m | none | none | none | | 0 |
| 19 | 48 | f | none | none | none | | 0 |
| 20 | 15 | f | 10-50 | none | none | y | 0 |
| 21 | 61 | m | none | 250 | 2 | | 0 |
| 22 | 21 | f | 1000 | 10-50 | none | | 0 |
| 23 | 40 | f | none | 10 to 50 | none | | 0 |
| 24 | 21 | f | none | none | 1 | | 0 |
| 25 | 31 | f | none | none | none | | 0 |
| 26 | 51 | m | 10 to 50 | 250 | 1 | | 0 |
| 27 | 38 | f | 10 to 50 | none | none | | 0 |
| 28 | 45 | m | none | 800 | none | | 0 |
| 29 | 18 | m | none | none | none | | nd |
| 30 | 42 | m | 3 to 9 | 10 to 50 | none | | 1-99 |
| 31 | 29 | m | none | none | >2 | | 1-100 |
| 32 | 52 | m | 10 to 50 | 10 to 50 | 1 | | 50-500 |
| 33 | 63 | f | 10-50 | 1000 | 1 | | 1-99 |
| 34 | 43 | f | none | 100 | none | | nd |
| 35 | 15 | m | 200 | 3 to 9 | 1 | | 1-99 |
| 36 | 25 | f | 10-50 | 3-9 | >2 | | 1-99 |
| 37 | 19 | m | 2 | 10 to 50 | 1 | | 1-99 |
| 38 | 36 | m | none | 150 | none | | nd |
| 39 | 30 | m | 10-50 | none | none | y | 1-99 |
| 40 | 43 | m | 10-50 | 10-50 | 1 | | 1-99 |
| 41 | 3 | m | 10 to 50 | 10 to 50 | 1 | | 1-99 |
| 42 | 44 | m | 100 | 3000 | none | | 1-99 |
| 43 | 62 | f | 3 to 9 | 500 | 2 | | 1-99 |

| | | | | | | | |
|---|---|---|---|---|---|---|---|
| 44 | 18 | f | 10-50 | none | none | | 1-99 |
| 45 | 24 | m | 10-50 | none | 2 | | 1-99 |
| 46 | 30 | f | none | 10-50 | none | | nd |
| 47 | 40 | m | none | 10-50 | 1 | y | 1-99 |
| 48 | 40 | m | 70 | 100 | 1 | | 1-99 |
| 49 | 22 | f | 10-50 | 3 to 9 | 2 | | 1-99 |
| 50 | 46 | m | 10 to 50 | 400 | >2 | | 1-99 |
| 51 | 18 | f | 2 | none | >2 | y | 1-99 |
| 52 | 14 | m | none | 10-50 | none | | nd |
| 53 | 35 | f | 3-9 | none | none | y | 100-500 |
| 54 | 9 | f | 10-50 | 2 | 1 | y | 100-500 |
| 55 | 29 | f | 120 | 1000 | none | y | >500 |
| 56 | 30 | f | 10 to 50 | 200 | >2 | | 100-500 |
| 57 | 28 | m | 10-50 | 3-9 | none | y | 100-500 |
| 58 | 29 | f | 200 | 400 | 2 | y | >500 |
| 59 | 36 | m | none | none | none | | nd |
| 60 | 37 | m | 10 to 50 | 1000 | >2 | y | 100-500 |
| 61 | 20 | f | 10 to 50 | 3 to 9 | 1 | | 100-500 |
| 62 | 40 | m | 150 | 10 to 50 | >2 | y | 100-500 |
| 63 | 26 | f | 3-9 | 10-50 | 2 | y | 100-500 |
| 64 | 44 | f | 60 | 10 to 50 | none | | 100-500 |
| 65 | 29 | f | 10 to 50 | 10-50 | none | y | 100-500 |
| 66 | 25 | f | 3 to 9 | 10 to 50 | 1 | | 100-500 |
| 67 | 39 | m | none | 3-9 | 1 | | 100-500 |
| 68 | 26 | m | 1200 | 100 | 2 | y | >500 |
| 69 | 40 | f | 70 | 2000 | >2 | | 100-500 |
| 70 | 41 | m | 100 | 3-9 | >2 | y | 100-500 |
| 71 | 63 | f | 10 to 50 | 200 | >2 | y | 100-500 |
| 72 | 51 | m | none | 1000 | >2 | y | 100-500 |
| 73 | 31 | f | 1000 | 1000 | >2 | | 100-500 |
| 74 | 60 | m | 3-9 | 300 | 1 | y | 100-500 |
| 75 | 39 | f | 100 | 300 | 1 | y | 100-500 |
| 76 | 30 | f | none | 3-9 | 2 | | 100-500 |
| 77 | 17 | m | 1000 | 500 | 1 | y | >500 |
| 78 | 17 | m | 2000 | none | none | y | >500 |
| 79 | 51 | m | 60 | 10-50 | >2 | y | >500 |
| 80 | 27 | f | 700 | none | 2 | y | >500 |
| 81 | 18 | m | 3 to 9 | 2 | 1 | | >500 |
| 82 | 26 | f | 10 to 50 | 800 | 1 | y | >500 |
| 83 | 18 | f | 10-50 | 10-50 | 2 | | >500 |
| 84 | 22 | m | 3-9 | 3-9 | 1 | y | >500 |
| 85 | 15 | f | 3 to 9 | 3 to 9 | 2 | y | >500 |
| 86 | 38 | m | 3 to 9 | none | >2 | y | >500 |
| 87 | 37 | m | 10-50 | 200 | >2 | | >500 |
| 88 | 45 | f | 100 | 3000 | 2 | y | >500 |
| 89 | 23 | m | 2000 | 1000 | >2 | | >500 |
| 90 | 16 | f | 2 | 10 to 50 | 2 | | nd |

| | | | | | | | |
|---|---|---|---|---|---|---|---|
| 91 | 45 | f | none | >50 | 1 | | nd |
| 92 | 32 | f | none | 1000 | 1 | | nd |
| 93 | 16 | m | none | 3 to 9 | >2 | | nd |
| 94 | 40 | f | 60 | 250 | 1 | | nd |
| 95 | 13 | m | 2 | none | 2 | | nd |
| 96 | 32 | f | 3 to 9 | none | none | | nd |
| 97 | 28 | f | 10-50 | 100 | >2 | | nd |
| 98 | 41 | f | none | >50 | none | | nd |
| 99 | 45 | m | 10-50 | 100 | none | | nd |
| 100 | 50 | m | 3-9 | none | none | | nd |
| 101 | 20 | f | 2 | 2 | none | | 1-99 |
| 102 | 32 | m | 3-9 | none | none | | 0 |
| 103 | 25 | m | none | 3-9 | none | | 0 |
| 104 | 23 | m | none | 10 to 50 | >2 | y | 100-500 |
| Healthy Control Group | | | | | | | |
| 1 | 53 | w | | | | | |
| 2 | 48 | w | | | | | |
| 3 | 48 | w | | | | | |
| 4 | 47 | w | | | | | |
| 5 | 42 | w | | | | | |
| 6 | 64 | w | | | | | |
| 7 | 44 | w | | | | | |
| 8 | 28 | w | | | | | |
| 9 | 73 | w | | | | | |
| 10 | 24 | w | | | | | |
| 11 | 67 | m | | | | | |
| 12 | 37 | m | | | | | |
| 13 | 58 | m | | | | | |
| 14 | 53 | m | | | | | |
| 15 | 25 | m | | | | | |
| 16 | 28 | m | | | | | |
| 17 | 29 | m | | | | | |
| 18 | 28 | m | | | | | |
| 19 | 38 | m | | | | | |
| 20 | 38 | m | | | | | |
| 21 | 41 | w | | | | | |
| 22 | 63 | w | | | | | |
| 23 | 66 | w | | | | | |
| 24 | 39 | w | | | | | |
| 25 | 47 | w | | | | | |
| 26 | 63 | w | | | | | |
| 27 | 61 | w | | | | | |
| 28 | 30 | w | | | | | |
| 29 | 34 | w | | | | | |
| 30 | 28 | w | | | | | |
| 31 | 56 | w | | | | | |
| 32 | 60 | w | | | | | |

| | | | | | | | |
|---|---|---|---|---|---|---|---|
| 33 | 40 | m | | | | | |
| 34 | 61 | m | | | | | |
| 35 | 55 | m | | | | | |
| 36 | 33 | m | | | | | |
| 37 | 31 | m | | | | | |
| 38 | 56 | m | | | | | |
| 39 | 57 | m | | | | | |
| 40 | 64 | m | | | | | |
| 41 | 57 | m | | | | | |
