# Supplementary material for: Serum biomarkers for neurofibromatosis type 1 and early detection of malignant peripheral nerve-sheath tumors
Source: BMC Med. 2013 Apr 23;11:109. doi: 10.1186/1741-7015-11-109 (PMC3648455; doi:10.1186/1741-7015-11-109)
Supplement: Additional file 2 — List of candidate markers selected by manual curation of published data and text. The proteins used in the screenings are shown in bold and italic [49-63]. [file 1741-7015-11-109-S2.pdf]

Additional file 2: List of candidate markers selected by manual curation of published data and text.

	abbr.	protein/ factor	PMID	immuno-modula-tory	tumori genesis
1	ADA	adenosine deaminase	[49]		x
2	ADM	adrenomedullin	[23]		x
3	***ANG-1***	angiopoietin-1	[50]		x
4	***Ast***	angiostatin	[62]		x
5	***BDNF***	brain-derived neurotrophic factor	[62]		x
6	bFGF	basic fibroblast growth factor	[63]		x
7	BLBP	brain lipid-binding protein	[51]		x
8	BMP1	bone morphogenetic protein 1	[52]		x
9	BMP2	bone morphogenetic protein 2	[62]		x
10	CCND1	cyclin D1 protein	[62]		x
11	CD9	cluster of differentiation 9	[62]	x	
12	***CD40***	cluster of differentiation 40	[63]	x	
13	***CD40L***	cluster of differentiation 40L	[63]	x	
14	CDH 19	cadherin 19, type 2	[63]		x
15	CDH 2	n-cadherin (neural)	[63]		x
16	CDH 3	P-cadherin (placental)	[62]		x
17	CDH11	cadherin 11 (OB-cadherin, Osteoblast)	[62]		x
18	CDH12	cadherin 12 (N-Cadherin, type2)	[62]		x
19	CIII	collagen type III	[63]		x
20	CVIa3	collagen type VIa3	[49]		x
21	CVIIa1	collagen type VIIa1	[49]		x
22	***CX3CL1***	fractalkine	[63]	x	
23	CXI	collagen type XI	[62]		x
24	EDN1	endothelin	[63]		x
25	***EGF***	epidermal growth factor	[62]		x
26	***EGF R***	epidermal growth factor receptor	[50]		x
27	FAS	FAS	[52]		x
28	FAS R	FAS receptor	[62]		x
29	FGF R2	fibroblast growth factor receptor 2	[52]		x
30	FGF R3	fibroblast growth factor receptors 3	[52],[50]		x
31	FGF R4	fibroblast growth factor receptors 4	[52]		x
32	***FGF1***	fibroblast growth factor acidic	[62]		x
33	***FGF2***	fibroblast growth factor 2 (basic)	[50]		x
34	FGF4	fibroblast growth factor 4	[52]		x
35	FGF5	fibroblast growth factor 5	[63]		x
36	***FGF7***	fibroblast growth factor 7	[50]		x
37	FGFR-7	fibroblast-growth-factor receptor 7	[62]		x
38	FN	fibronectin	[62]		x
39	***GDNF***	*glial cell line-derived neurotrophic factor*	[53]		x
40	Gli1	glioma-associated oncogene homolog 1	[54]		x

	abbr.	protein/ factor	Source	immuno-modula-tory	tumori genesis
41	***GROa***	growth-regulated alpha protein	[50]		x
42	HGF	hepatocyte growth factor	[50]		x
43	***I-309***	I-309	[62]	x	
44	***IFNg***	*interferon, gamma*	[55]	x	
45	IGF-1R	insulin-like growth factor receptor 1	[52]		x
46	***IGF2***	insulin-like growth factor 2	[52]		x
47	IGF-2R	insulin-like growth factor receptor 2	[52]		x
48	***IGFBP-1***	insulin-like growth factor-binding protein 1	[56]		x
49	***IGFBP-2***	insulin-like growth factor-binding protein 2	[62]		x
50	***IGFBP3***	insulin-like growth factor binding protein 3	[62]		x
51	***IGFBP-4***	*insulin-like growth factor-binding protein 4*	[57]		x
52	***IGFBP-6***	*insulin-like growth factor binding protein 6*	[49]		x
53	***IGF-I***	insulin-like growth factor 1	[52]		x
54	***IL-10***	*interleukin 10*	[62]	x	
55	***IL-12 p70***	*interleukin 12p70*	[62]	x	
56	***IL-12p40***	interleukin 12p40	[62]	x	
57	***IL-1b***	*interleukin 1b*	[52]	x	
58	IL-3	interleukin 3	[52]	x	
59	IL-3R	interleukin 3 receptor	[52]	x	
60	***IL-4***	*interleukin 4*	[29]	x	
61	***IL-6***	*interleukin 6*	[58]	x	
62	***IL-8***	*interleukin 8*	[50]	x	
63	***IP-10***	*interferon gamma-induced protein 10*	[62]	x	
64	ITG alpha V	integrin alpha V (CD51)	[52],[50]	x	
65	ITGB 1	integrin β1 (CD29)	[52]		x
66	KRT18	cytokeratin 18	[49]		x
67	LIF	leukemia inhibitory factor	[62]		x
68	***MCP-1***	*monocyte chemotactic protein-1*	[62]	x	
69	MIA	melanoma inhibitory activity	[21]		x
70	***MIF***	macrophage migration inhibitory factor	[62]	x	
71	***MIG***	*monokine induced by IFNγ*	[62]	x	
72	MK	*midkine*	[20]		x
73	MMP9	*matrix metalloproteinase-9*	[54]		x
74	MMP13	matrix metalloproteinase-13	[59]		x
75	***MPIF-1***	myeloid progenitor inhibitory factor-1	[62]	x	
76	***NAP-2***	neutrophil-activating protein-2	[62]	x	
77	NCAM (CD56)	neural cell adhesion molecule	[62]	x	
78	NRP1	neuropilin-1	[50]		x
79	***NGF R***	nerve growth factor receptor	[62]		x
80	***OPG***	osteoprotegerin	[63]	x	

	abbr.	protein/ factor	Source	immuno-modula-tory	tumori genesis
81	OPN	osteopontin	[50]		x
82	***PARC (MIP-2)***	pulmonary and activation-regulated chemokine	[63]	x	
83	PDGF-AB	platelet-derived growth factor AB	[52],[50]		x
84	***PDGF-BB***	*platelet-derived growth factor BB*	[63]		x
85	PDGFRa	platelet-derived growth factor receptor a	[50]		x
86	PlGF	placenta growth factor	[50]		x
87	***RANTES***	regulated upon activation, normal T-cell expressed, and secreted	[62]	x	
88	SCF	stem cell factor	[20]		
89	***SDF-1***	*stromal cell-derived factor-1*	[62]		x
90	***sgp130***	soluble glucoprotein 130	[63]	x	
91	***ShhN***	sonic hedgehog	[54]		x
92	SIVA-1	CD27-binding (Siva) protein	[62]	x	
93	SPARC	secreted protein acidic and rich in cysteine	[50] ,[52]		x
94	survivin	survivin	[49]		x
95	***TGFß1***	transforming growth factor beta 1	[52]	x	
96	***TGFα***	transforming growth factor α	[63]	x	
97	***Tie-1***	tyrosine kinase with immunoglobulin-like and EGF-like domains 1	[62]	x	
98	***Tie-2***	tyrosine kinase with immunoglobulin-like and EGF-like domains 2	[62]	x	
99	***TIMP-1***	tissue inhibitor of matrix metalloproteinase 1	[50]		x
100	***TIMP-2***	tissue inhibitor of matrix metalloproteinase 2	[50]		x
101	TNC	tenascin C	[49],[60]		x
102	***TNF-α***	tumor necrosis factor α	[50],[52]	x	
103	TNF-R1	tumor necrosis factor α receptor	[52]	x	
104	TNXB	tenascin XB	[60]		x
105	***TRAIL R2***	TNF-related apoptosis-inducing ligand receptor 2	[54]		x
106	***TRAIL R3***	TNF-related apoptosis-inducing ligand receptor 3	[63]		x
107	***TRAIL R4***	TNF-related apoptosis-inducing ligand receptor 4	[63]		x
108	TSP-1	thrombospondin 1	[63]		x
109	TYMS	thymidylate synthase	[63]		x
110	***u PAR***	urokinase receptor	[50]		x

	abbr.	protein/ factor	Source	immuno-modula-tory	tumori genesis
111	uPA	urokinase	[46]		**x**
112	***VCAM-1***	vascular cell adhesion protein 1	[61]	**x**	
113	***VEGF***	vascular endothelial growth factor	[44]		**x**
114	VEGFR3	vascular endothelial growth factor receptor 3	[52]		**x**
115	WWOX	WW containing oxidoreductase	[59]		**x**
