# Supplementary material for: Serum biomarkers for neurofibromatosis type 1 and early detection of malignant peripheral nerve-sheath tumors
Source: BMC Med. 2013 Apr 23;11:109. doi: 10.1186/1741-7015-11-109 (PMC3648455; doi:10.1186/1741-7015-11-109)
Supplement: Additional file 3 — Reassessment of protein serum markers interferon (IFN)-γ, interleukin (IL)-6, tumor necrosis factor (TNF)-α, insulin-like growth factor binding protein (IGFBP) and Regulated upon activation, normal T-cell expressed and secreted (RANTES) by cytometric bead array (CBA) and ELISA (arbitrary serum concentration units). Between 11 and 15 randomly selected sera from the different NF1 groups (for IFN-γ, IL-6, TNF-α: all NF1 vs. control; for IGFBP and RANTES: NF1 with no PNF or MPNST, NF1 with only PNF- and NF1 with MPNST) and 5 control sera were tested as indicated (ND, not determined). Where available, associated protein array data are shown. Statistical analysis is shown for CBA/ELISA data (t-test). [file 1741-7015-11-109-S3.pdf]

Additional file 2: Re-assessment of protein serum markers IFNγ, IL6, TNFα, IGFBP and Rantes by CBA and ELISA (arbitrary serum concentration units). Between 11 and 15 randomly selected sera from the different NF1 groups (For IFNγ, IL6, TNFα: all NF1 vs. control; for IGFBP and Rantes: NF1 with no PNF or MPNST, NF1 with only PNF and NF1 with MPNST) and 5 control sera were tested as indicated (n.d. not determined). Where available, associated protein array data are shown. Statistical analysis is shown for Array and CBA/ELISA data (T-Test).

No	ID	Age	Gender	IFNγ		IL6		TNFα			IGFBP1				Rantes			
				Array	CBA	Array	CBA	Array	CBA		Array		ELISA		Array		CBA	
1	Control	53	f	102	43,2	455	3416,1	748,2	8,2		16533,3		190,6		36901,5		17761,8	
2	Control	31	m	166	46,5	355	3322,8	572	7,4		9376		80,6		33597		22715	
3	Control	25	m	102	48,9	227,2	3356,3	363,3	8,5		4401		87,5		31793,4		32177,4	
4	Control	38	f	n.d.	34,8	n.d.	3495,6	n.d.	7,8		n.d.		234,4		n.d.		21001,8	
5	Control	58	m	n.d.	38,8	n.d.	3559,4	n.d.	6,7		n.d.		160,5		n.d.		16461,2	
6	Control	28	m	n.d.	n.d.	n.d.	404,1	n.d.	n.d.		n.d.		n.d.		n.d.		n.d.	
7	Control	64	f	47,7	n.d.	225,1	401,4	289,3	n.d.		1377,6		n.d.		36452,3		n.d.	
	Average	42		104,43	**42,44**	315,58	**2565,1**	493,2	**7,72**		7921,98		**150,72**		34686,1		**22023,44**	
1	NF1	20	f	216,1	41,3	529,3	3373,6	921,9	n.d.		14546,8		220,2		28500,6		25158,4	
2	NF1	26	m	218,5	52,8	472	3744,8	859,9	8,3		17672,3		210,1		36566,7		43956,3	
3	NF1	14	m	191,7	55,2	551,8	3451,1	972,6	n.d.		17968,1		166,7		35254,1		45736,9	
4	NF1	41	f	215,2	58,5	779,7	3719,4	1180,4	8,7		10462		193,8		33562,2		26864,4	
5	NF1	46	m	139,4	52,8	285,2	3802,8	439,3	n.d.		19422		428,3		32496,2		21692,5	
6	NF1	31	m	313	57,6	568	4039,2	972	9,6		25915		199,4		53085		18748,8	
7	PNF	51	m	289,3	n.d.	628,8	3931,2	823,8	8,7		12079,9		133,4		29294,3		44394,1	
8	PNF	40	m	134	48,1	437	3844,2	524	8		21106		371,1		29225		17445,7	
9	PNF	44	f	n.d.	n.d.	n.d.	2719,4	n.d.	n.d.		n.d.		n.d.		n.d.		n.d.	
10	PNF	36	m	137,3	54,4	398,4	3390,3	597,1	8,5		13030,1		221,4		40942		14736,7	
11	PNF*high tumor load	18	m	207	52	546,2	3568,6	870,8	8,4		23680,2		1305,9		28649,3		26613,1	
12	MPNST	26	m	896	54,4	1070	2719,4	2855	8,3		32491		430,2		49833		75390,3	
13	MPNST	38	m	212	55,2	325	n.d.	950	n.d.		24079		772,2		37519		47836,2	
14	MPNST	40	f	216	n.d.	488,4	n.d.	784	8,7		21849,9		342,8		27270,7		45284,4	
15	MPNST	39	f	247	n.d.	637	n.d.	1019	8,6		22567		191,3		31883		63863,2	
16	MPNST	43	f	284,4	n.d.	569,5	n.d.	1072,4	8,3		29761,5		1086,5		46496,6		52461,3	
	Average	35		261,13	**52,936**	552,42	**3525,3**	989,48	**8,5545**	**NF1**vs control	17664,4	p<0.031	**236,417**	p<0.13	36577,5	p<0.69	**30359,55**	p<0.19
	NF1 vs control			P<0.116	p<0.002	p<0.031	p<0.05	p<0.103	p<0.01	**PNF** vs control	17474,1	p<0.073	**507,95**	p<0.181	32027,7	p<0.44	**25797,4**	p<0.59
										**MPNST** vs control	26149,7	p<0.002	**564,6**	p<0.036	38600,5	p<0.46	**56967,08**	p<0.01
